# A combined treatment regimen of MGMT-modified γδ T cells and temozolomide chemotherapy is effective against primary high grade gliomas

**DOI:** 10.1038/s41598-021-00536-8

**Published:** 2021-10-26

**Authors:** Lawrence S. Lamb, Larisa Pereboeva, Samantha Youngblood, G. Yancey Gillespie, L. Burton Nabors, James M. Markert, Anindya Dasgupta, Catherine Langford, H. Trent Spencer

**Affiliations:** 1 Department of Medicine, Division of Hematology and Oncology, University of Alabama at Birmingham, Birmingham, AL USA; 2 Department of Neurosurgery, University of Alabama at Birmingham, Birmingham, AL USA; 3 Department of Neurology, Division of Neuro-Oncology, University of Alabama at Birmingham, Birmingham, AL USA; 4 Department of Pediatrics, Aflac Cancer and Blood Disorders Center, Emory University, Atlanta, GA USA

**Keywords:** Applied immunology, Cell delivery, Gene therapy, Tissue engineering, Cancer, Immunology, Oncology

## Abstract

Chemotherapeutic drugs such as the alkylating agent Temozolomide (TMZ), in addition to reducing tumor mass, can also sensitize tumors to immune recognition by transient upregulation of multiple stress induced NKG2D ligands (NKG2DL). However, the potential for an effective response by innate lymphocyte effectors such as NK and γδ T cells that recognize NKG2DL is limited by the drug’s concomitant lymphodepleting effects. We have previously shown that modification of γδ T cells with a methylguanine DNA methyltransferase (MGMT) transgene confers TMZ resistance via production of O^6^-alkylguanine DNA alkyltransferase (AGT) thereby enabling γδ T cell function in therapeutic concentrations of TMZ. In this study, we tested this strategy which we have termed Drug Resistant Immunotherapy (DRI) to examine whether combination therapy of TMZ and MGMT-modified γδ T cells could improve survival outcomes in four human/mouse xenograft models of primary and refractory GBM. Our results confirm that DRI leverages the innate response of γδ T cells to chemotherapy-induced stress associated antigen expression and achieves synergies that are significantly greater than either individual approach.

## Introduction

Gamma/delta (γδ) T cells are a specialized population of T cells with a restricted T-cell receptor (TCR) that is made up of one γ (gamma) chain and one δ (delta) chain. There γδ T cells combine innate and adaptive immune properties and contribute to tumor immunosurveillance^1^ through detection of stress-induced proteins, phosphoantigen/butyrophilin 3A1, CD1c, CD1d, inflammatory cytokines, and/or other stress/danger signals. Surface NKG2DL such as MHC class I polypeptide-related sequence A and B (MIC-A, MIC-B) and UL-16 Binding Proteins (ULBP) bind to both NKG2D and to the γδ T cell receptor leading to perforin and granzyme-mediated killing, release of proinflammatory cytokines, and potentially the priming of a CD8 + αβ T cell response ^2^ as a result of the adaptive functions of Vγ9Vδ2 + T cells.

Several recent studies of solid extracranial cancers have shown that strategic timing of chemotherapy and immunotherapy can achieve synergies that are significantly greater than either individual approach ^3–8^. In addition to reducing tumor mass, thereby reverting T cell exclusion and promoting T cell infiltration^9^, chemotherapy can also generate pro-inflammatory T cell and myeloid cytokine expression^10^. Alkylating agents such as Temozolomide (TMZ), the primary chemotherapeutic agent for glioblastoma (GBM), methylates the O^6^ position of guanine residues thereby inducing mispairing with thymine during replication inducing cascading cycles of thymine excision and reinsertion, DNA strand breaks, and activation of the DNA damage response leading to cytotoxicity. Most importantly, these initial mispairing events can result in transient but significant upregulation of Natural Killer Group 2D Ligands (NKG2DL) even in TMZ-resistant tumor cells thereby creating a window of vulnerability through this transiently increased density of target ligands for γδ T cells^11,12^ and activation of intrinsic mitochondrial pathway of apoptosis via p53, P-21, and ɣ-H2AX activation^13^.

Unfortunately, the lymphodepleting properties of chemotherapy dampen an effective cellular immune response at the time of the tumor’s greatest vulnerability^12,14^. To counter this problem, we have previously shown that resistance to alkylating agents can be conferred to T cells and NK cells by genetically modifying these effectors to express AGT from an MGMT transgene^12,15,16^ allowing full function at therapeutic concentrations of chemotherapy. Based on these observations, we developed a treatment strategy we have termed Drug Resistant Immunotherapy (DRI) for high-grade gliomas such as Glioblastoma Multiforme (GBM). This approach incorporates TMZ–induced upregulation of NKG2DL expression and simultaneous targeting with MGMT-modified γδ T cells.

## Results

### Graft characteristics

**Figure 1:**
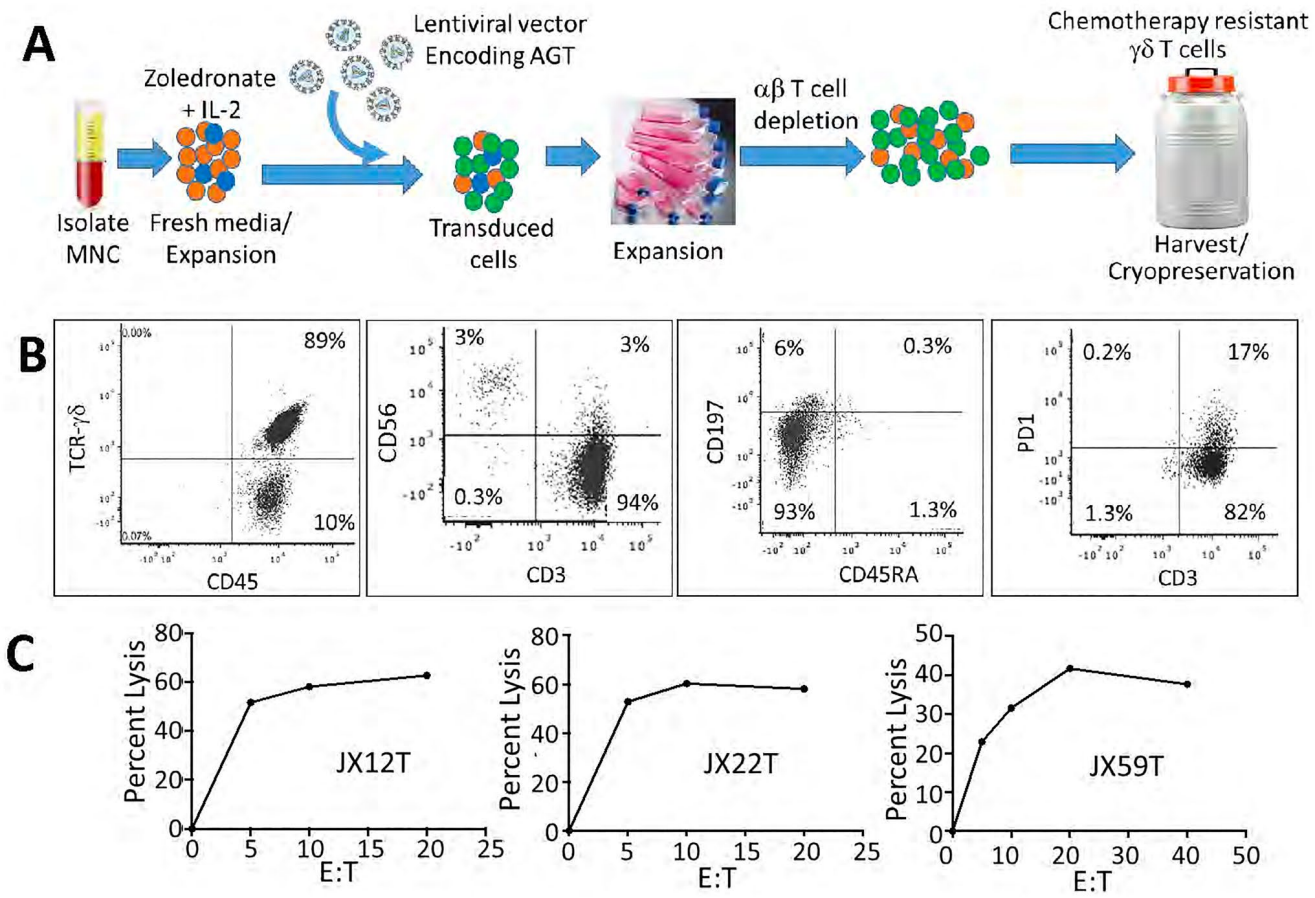
DRI product manufacturing protocol and phenotypic and functional characteristics of MGMT-modified and ex vivo expanded γδ T cells. (**A**) Following density gradient centrifugation, a 5 mL aliquot of apheresis product from a healthy volunteer is placed into culture media containing zoledronic acid and IL2 and incubated with P140K-MGMT lentiviral vector at an MOI of 10 on the fifth and sixth day of culture. The cell product is harvested when the γδ T-cell expansion plateaus, usually between days + 10 to + 16, αβ T cells are depleted if the γδ T cell content is < 70%, and the expanded cells are then cryopreserved for subsequent thawing and administration. (**B**) The final cell product contains 70–90% γδ T cells with a minimal component of αβ T cells and NK cells. Expanded γδ T cells exhibit effector/memory phenotype and minimal PD-1 expression and (**C**) show strong cytolytic activity against TMZ-resistant PDX cell lines JX12T and mesenchymal subtypes JX22T and JX59T following 4 h of co-incubation at 37 °C.

The DRI therapeutic agent was manufactured using a modified version of a previously described method that activates circulating Vγ9Vδ2 T cells using a combination of Zoledronic acid (Zoledronate) and IL-2 (Fig. 1A). The γδ T cells are transduced during culture with a P140K-MGMT expressing lentivirus. Cultured cell products obtained using this method are almost exclusively lymphocytes containing ~ 80–90% γδ T cells, < 10% residual αβ T cells, and generally < 5% NK cells (Fig. 1B). The MGMT-modified γδ T cells are predominately effector/memory phenotype with minimal upregulation of PD-1. Cultured γδ T cells were cytotoxic in vitro to TMZ-resistant GBM xenolines in a dose-dependent manner (Fig. 1C).

### Effect of monotherapies, combination therapies, and timing on disease progression and survival

**Figure 2:**
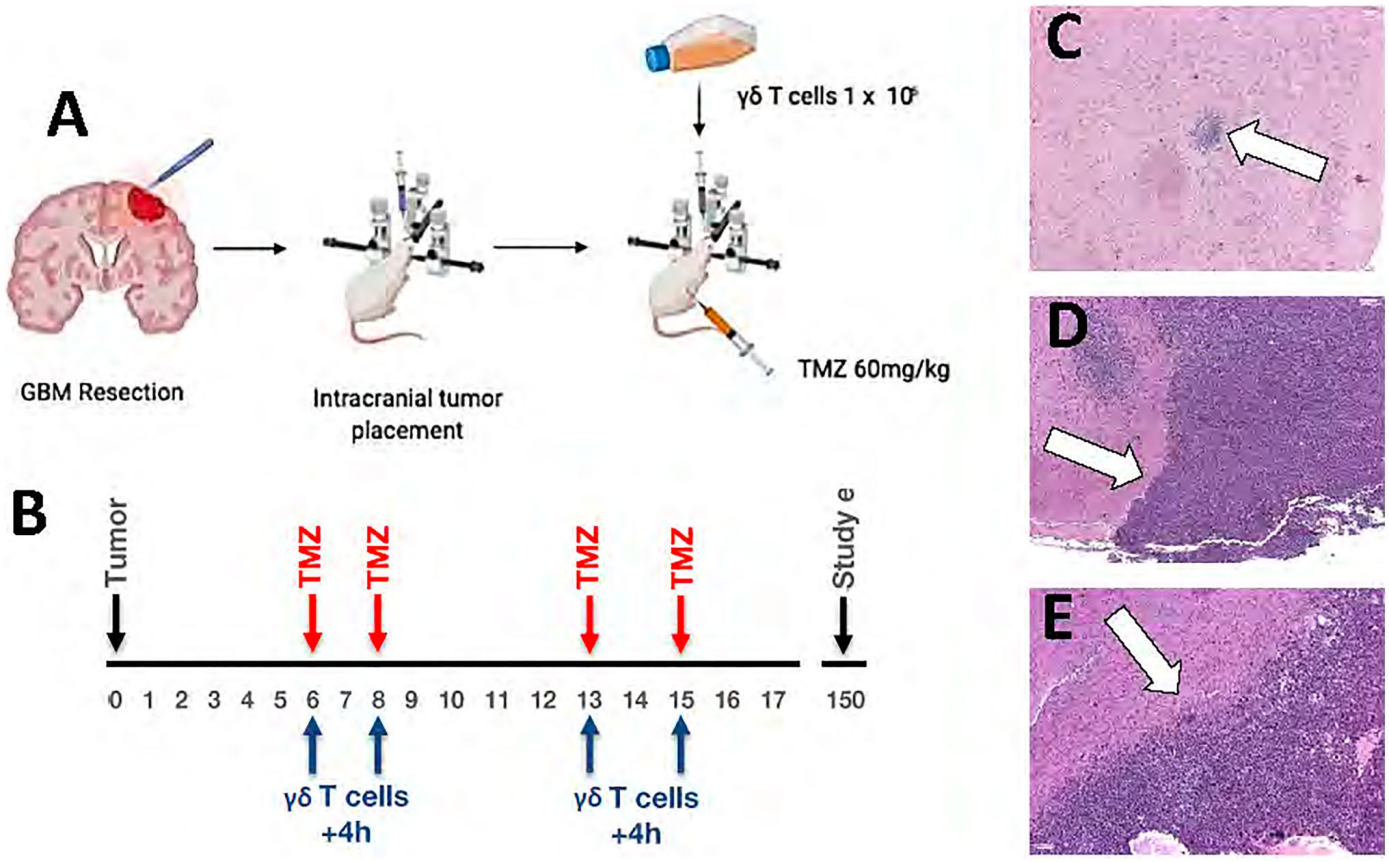
Study protocol. (**A**) Single-cell disaggregated PDX tumors are prepared from primary and TMZ-resistant GBM and were injected into the left cerebral hemisphere using a stereotactic frame. Mice receiving MGMT-modified γδ T cells received intracranial injections using the identical stereotactic coordinates. MGMT was delivered intraperitoneally Adapted from “General Figures”, by BioRender.com (2021). Retrieved from https://app.biorender.com/biorender-templates. (**B**) Tumor-bearing mice received two cycles intraperitoneal (IP) 60 mg/kg TMZ and 1 × 10^6^ MGMT-modified γδ T cells concurrently spaced 48 h apart and repeated one week later. Mice treated with single-agent TMZ or γδ T cells maintained the same schedule for the respective agent. (**D**) Hematoxylin and Eosin (H&E) staining of digital light microscopic image (4 ×) of an untreated JX12P PDX tumor (white arrows) at post-injection day + 6 and (**D**) the characteristic rapid growth of the tumor at day + 16. Image (**E**) shows a 16-day tumor after four cycles of TMZ chemotherapy. Note that although significant damage to the tumor has occurred several viable and proliferating colonies remain.

**Figure 3:**
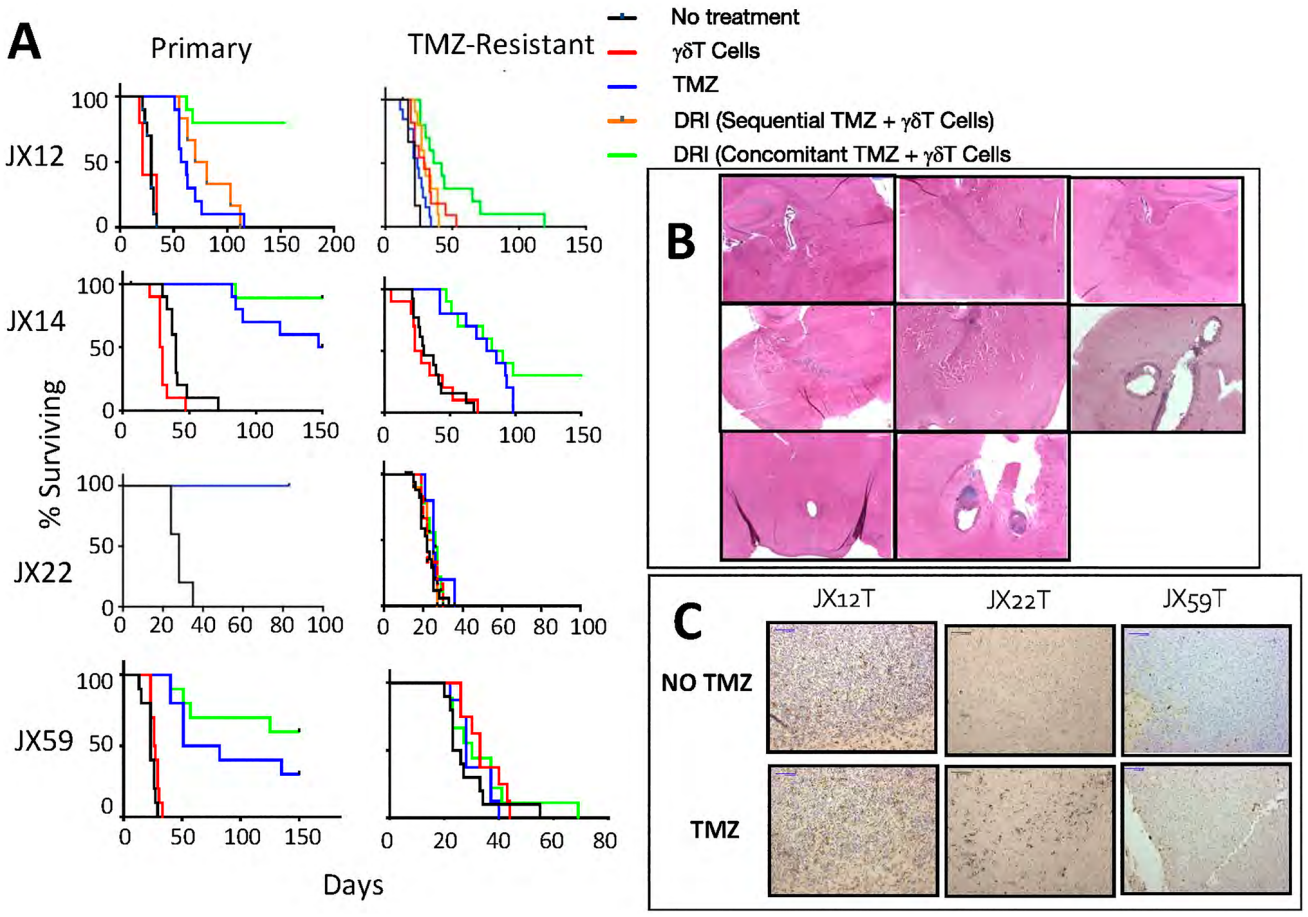
Response of an immunodeficient mouse model of intracranial PDX to Drug Resistant Immunotherapy. (**A**) Survival curves for mice that received one of four primary or TMZ resistant PDX. Groups include no treatment (black), single-agent MGMT-modified γδ T cells (red), single-agent TMZ (blue) and DRI (green). (**B**) H&E-stained whole-brain coronal sections through the injection site from all eight DRI-treated surviving JX12 mice at + 150 days post-tumor injection. At the time of euthanasia, these mice showed no neurologic dysfunction and no histologic evidence of residual tumor. (**C**) PD-L1 expression for TMZ-resistant tumors JX12T, JX22T, and JX59T from untreated mice and mice that received four cycles of single-agent TMZ showing increased PD-L1 expression in TMZ-treated tumors.

Intracranial tumors were established in athymic nude mice (*n* = 10/group) from human primary (P) or TMZ-resistant (T) JX12 and JX14 (classical) or JX22 and JX59 (mesenchymal) patient derived xenolines (PDX) using stereotactic injections of 1 × 10^5^ cells (Fig. 2A), a dose that is calibrated for an overall survival of 30–40 days. In a stringent test of DRI, 1 × 10^6^ intracranial MGMT-modified γδ T cells were administered within 4 h of intraperitoneal (IP) 60 mg/kg TMZ (Fig. 2b). Tumors in untreated mice progressed from a small organizing mass on Day + 6 (Fig. 2c) and expanded rapidly during the treatment window (Fig. 2d). Untreated mice (black lines, all graphs) exhibited severe neurologic dysfunction requiring euthanasia for the entire cohort by Day + 35 post-tumor placement (Fig. 3a). Four cycles of single-agent TMZ therapy was effective at achieving some tumor lysis (Fig. 2e), slowing symptomatic progression in some animals, and improving survival in mice with primary tumors (Fig. 3a). The classical primary GBM JX22P-bearing cohort that received single-agent TMZ therapy all survived to the study endpoint and although small tumors were present at Day + 150 in most mice, this group was not evaluated further for DRI response. As expected, single-agent TMZ therapy did not extend survival for any cohort bearing TMZ-resistant tumors. Treatment with four cycles of single-agent MGMT-modified γδ T cells did not provide a survival advantage for any TMZ-resistant PDX-bearing cohort.

The DRI combination significantly improved survival over single agent TMZ for mice bearing the classical primary JX12P GBM (*p* < 0.001) and mesenchymal primary JX59P (p = 0.044) as well as the classical TMZ-resistant tumor JX12T (p = 0.017). Mice bearing the classical primary JX14P tumor also showed a trend toward improvement (p = 0.0858) and although it’s TMZ-resistant counterpart showed no improvement in median survival over TMZ, three long-term tumor-free survivors were noted (Fig. 3a). Finally, we also showed that TMZ-resistant mesenchymal GBM JX59T and JX22T tumors did not respond to DRI. In all groups, DRI-treated mice that survived to Day + 150 showed no histopathologic evidence of recurrent GBM (Fig. 3b JX12 shown). In contrast, mice that were euthanized due to neurologic impairment all showed large infiltrating glioblastoma tumors with high mitotic index and central necrosis. Taken together, these findings suggest that DRI does not impede γδ T cell function and improves survival for mice bearing primary GBM PDX.

### Effect of temozolomide on PD-L1 expression

Interestingly, we also observed a small but consistent upregulation of PD-L1 expression on TMZ-treated resistant PDX over mice that did not receive TMZ (Fig. 3c). These findings are consistent with previous studies describing the corruption of pattern recognition receptors (PRR), such as cGAS/STING, that occur in conjunction with the DNA-damaging effect of alkylating agents^17^ and subsequent upregulation of PD-L1, suggesting the potential for modulation of immune checkpoints PD-1 and PD-L1 as a strategy to improve efficacy of DRI.

## Discussion

This report is consistent with our previous work showing innate recognition of GBM as a potential therapeutic strategy^18,19^ and that cDNA sequences encoding chemotherapy resistance can protect immune cells from the damaging effects of cytoreductive drug therapy^16,20,21^. MGMT gene modification has been shown to protect hematopoietic progenitor cells (HPC) during chemotherapy regimens^16,20,21^, thereby reducing myelosuppression^22,23^ . This concept was evaluated in an early phase clinical trial in which MGMT-modified autologous HPC were transplanted into therapy-resistant GBM patients followed by dose-intensified BCNU chemotherapy. These patients survived longer than predicted without negative side effects and were able to tolerate high-dose chemotherapy better than did patients in previous studies who had received the same type of chemotherapy but without the gene-modified HPC. In a follow-on study, MGMT HPC modification increased in the mean number of tolerated O6BG/TMZ cycles but did not increase median progression-free survival or overall survival beyond historical controls.

Stress-associated surface proteins that are upregulated by TMZ and other alkylating agents promote immune responses that increase the potential for enhanced recognition^11,24^ and potentially the priming of adaptive immunity as a result of the antigen-presenting functions of Vγ9Vδ2 + T cells ^2^.

As expected, we observed increased median survival in primary PDX-bearing mice receiving TMZ chemotherapy. We used a dose and administration route for TMZ that allowed consistent dosing and predictable pharmacokinetics as opposed to the potential loss and absorption issues with oral gavage. Temozolomide prolonged median survival in all primary PDX groups tested, indeed, chemotherapy was curative in JX22P. Cell therapy alone did not result in a survival advantage for PDX-bearing mice even though their respective xenolines are readily killed in ex vivo cytotoxicity assays. This finding was consistent with our previous work showing that γδ T cell treatment alone, although cytotoxic in vitro to GL261 mouse glioma cells, failed to improve outcomes in a syngeneic intracranial treatment model^25^. Interestingly, the syngeneic model also revealed significant down-regulation of GL261-associated NKG2DL expression in a hypoxic environment more consistent with in vivo conditions. Taken together, this single-agent γδ T cell infusion approach is likely insufficient to overcome the heterogeneous nature, growth dynamics, and baseline down-regulation NKG2DL on the respective PDX lines (Fig. 1C).

The immune-sensitizing properties of chemotherapy and the potential synergies that can be achieved by combining chemotherapy with various immunotherapy regimens have been recently reviewed by others^11,26,27^. We previously showed that NKG2DL expression is significantly upregulated on TMZ-resistant GBM cell lines during the first 12 h following TMZ exposure and that a synergistic tumor killing effect could be achieved in vitro by combining simultaneous exposure of TMZ-resistant GBM cell lines to therapeutic concentrations of TMZ and MGMT-modified γδ T cells^12^. In addition, we reported TMZ-mediated NKD2DL upregulation on GBM xenolines *in vitro*^28^ (Figure S1a). Documentation of a pre/post TMZ upregulation of NKG2DL for intracranial PDXT was technically impractical due the challenges of serial pre/post TMZ stereotactic biopsies of a very small tumor in a mouse model, although we were occasionally able to capture this transition (Figure S1b–c). Improved survival in primary PDXT-bearing mice taken together with in vitro findings of increased cytotoxic activity and NKG2DL upregulation upon exposure to TMZ suggest that a similar mechanism may be partially responsible for the improved outcome in DRI-treated mice with primary tumors.

In practice, combination approaches can also result in effector cell activation and reduced accumulation of immunosuppressive cells, however, these effects on their own are not often sufficient to overcome immune barriers and induce effective clearance of disease and long-term remission. The effects of cytotoxic chemotherapy are also often globally lymphodepleting and therefore prevent an effective immune response when the tumor is at its most vulnerable state. One solution has been to delay immunotherapy until the drug concentration has fallen to a level that permits effector cell function but also timed to leverage the effects of tumor injury and suppressor cell depletion. Indeed, Nicol^29^ showed no beneficial effect of infusing autologous expanded and activated γδ T cells in patients with a variety of extracranial solid tumors except in cases when the infusion was given within 24 h of chemotherapy. Both in vitro and animal studies from our laboratory also suggest that vulnerability largely dissipates as the tumor recovers from chemotherapy-induced stress^12,28^. Additional immune stimulation induced by protection of γδ T effector lymphocytes from the cytotoxic effects of chemotherapy in the clinical setting may help overcome barriers to the establishment of long-term remission. Activated γδ T cells also stimulate DCs to mature and enhance antigen cross-priming^30^, upregulate costimulatory molecules, and attract naive CD8 + T cells. Combined with the potential of Vγ9Vδ2 + T cells to process and present antigen, γδ T cells could provide a mechanism for neoantigen presentation to CD8 + T cells and consequent epitope spreading.

Another advantage that chemotherapy provides is the clearance of immunosuppressive cells. Alkylating agents such as Temozolomide can deplete circulating and tumor-resident immunosuppressive CD11b^+^Gr-1^+^ monocyte derived suppressor cells (MDSCs) that inhibit antigen presentation and T Cell activation. through mechanisms that are incompletely described. MDSCs lack DNA repair proteins such as X-ray repair cross-complementing protein 1 and poly (ADP-ribose) polymerase 1, and are therefore vulnerable to alkylating agents^31^. Temozolomide treatment of human myeloid cells has also been shown to induce p53, P-21, and ɣ-H2AX activation, which prompts an intrinsic mitochondrial pathway of apoptosis^32^. However, in murine glioma models temozolomide has not been described to reduce macrophage derived chemokines. Additionally, low dose metronomic regimens of temozolomide, an alkylating agent, can reduce Treg to total CD4 + T lymphocyte ratios and impair Treg suppressive activity in glioma-bearing rats^33,34^.

We observed increased median survival with DRI principally in primary PDX-bearing mice. However, DRI was less effective in eradicating TMZ-resistant PDX and improving survival. Although we have shown that TMZ increases short-term surface expression of NKG2DL and improved sensitivity of TMZ-resistant GBM cell lines to γδ T cell-mediated lysis, several factors could impact on the cumulative effect of DRI on recurrent primary GBM. Chemotherapy alone has a significant effect on primary tumors, providing debulking and eradication of chemosensitive cells that comprise the majority of the tumor. With this debulking also comes a reduction in local immunosuppression, improved access to the tumor, and a more favorable effector-to-target ratio against TMZ-resistant cells.

In summary, we show that concurrent dosing of MGMT-modified γδ T cells can improve survival outcomes in a PDX model of both classical and mesenchymal subtypes of primary high-grade gliomas over either single-agent chemotherapy and single agent γδ T cell-based therapies. Our findings provide significant preclinical evidence supporting the development of Drug Resistant Immunotherapy for primary high-grade gliomas.

## Methods

### Mice

Athymic nude mice were all purchased from The Jackson Laboratory. All mice were maintained in pathogen-free facilities in the UAB Brain Tumor Animal Models (BTAM) Facility. This study was carried out in strict accordance with recommendations in the Guide for the Care and Use of Laboratory Animals of the National Institutes of Health and the ARRIVE guidelines. The protocol was approved by the Animal Care and Use Committee at the University of Alabama at Birmingham (Birmingham, AL). (APN20339). All surgery was performed under ketamine/xylazine anesthesia, and all efforts were made to minimize pain.

### Patients and apheresis donors

Patient-derived tumor xenografts (PDX) were a gift of David James to the UAB Brain Tumor Tissue Facility (BTTF) headed by G. Yancey Gillespie, PhD and included the primary classical lines JX12 and JX14, their TMZ-resistant pairs JX12T and JX14T, and the primary mesenchymal subtype pair JX22P and JX59 and TMZ-resistant JX22T and JX59T. Expanded and activated γδ T cells were manufactured from deidentified apheresis products obtained from healthy volunteers via Hemacare, Inc., a commercial blood products vendor and as such met the exemption criteria for the human subjects review committee at the University of Alabama at Birmingham.

### Intracranial injections

Intracranial gliomas were generated using 5 × 10^5^ human glioma xenolines suspended in 5% methylcellulose in serum-free medium using a previously reported method^18^. The cells were drawn into a 250 μl Hamilton gas-tight syringe mounted in a Chaney repeating dispenser and fitted with a 30G ½-inch needle with a calibrated depth of 2.5 mm from the middle of the bevel opening. Under an operating microscope, the fascia on the skull of the anesthetized mouse were scraped off and a 0.5 mm burr hole made 2 mm to the right of the midline suture and 1 mm caudal to the coronal suture. The syringe was inserted into a Kopf stereotactic electrode clamp mounting bracket attached to an electrode manipulator (David Kopf Instruments; Tujinga, CA) mounted on a Kopf stereotactic frame electrode A-P zeroing bar (#1450). Each mouse was positioned on the stereotactic frame and the needle inserted to the depth marker into the right cerebral hemisphere. Approximately 90–120 s after injection of 5 ml, the needle was slowly withdrawn over the next minute. The burr hole was plugged with sterile bone wax and skin is closed with Tissu-Mend surgical adhesive (Stryker Orthopedics; Kalamazoo, MI). Tumor engraftment was confirmed by assessment of luminescence. Control mice received tumor only, tumor + DRI cells only, or tumor + TMZ only. The major endpoint in this study was animal survival; moribund animals that became unresponsive to mild external stimuli were euthanized and this date was used as an estimate of the date of death.

### Treatment protocol

Tumor-bearing mice (*n* = 10/group) were treated twice weekly with intraperitoneal (IP) 60 mg/kg TMZ and intracranial (IC) of 1 × 10^6^ MGMT-modified γδ T cells within 4 h of the TMZ injections. Mice receiving cell therapy received stereotactic intracranial injections of γδ T cells approximating the original tumor placement at a dose of 1 × 10^6^ per injection. Chemotherapy-treated mice received 60 mg/kg Temozolomide intraperitoneally (IP). Tumor-bearing control mice were divided into three groups in which the first received IC MGMT-modified γδ T cells on days + 6, + 8, + 13, and + 15, the second received IP TMZ on days + 6, + 8, + 13, and + 15, and the third received no anti-tumor therapy. Survival was assessed using Kaplan–Meier analysis.

### Histopathology and immunohistochemistry

Sections of mouse brain with tumor were prepared and fixed in neutral buffered formalin in the Brain Tumor Tissue Facility of the UAB Comprehensive Cancer Center. There formalin-fixed paraffin-embedded (FFPE) sections were sectioned and stained with hematoxylin and eosin and examined for size, infiltration, and histologic grade. The level of expression of checkpoint and stress-induced markers in whole mouse brain and tumor were assessed by immunohistochemistry. As previously described^25^, deparaffinized sections were post fixed in 4% neutral buffered formalin followed by antigen retrieval with Citra Plus (Biogenex Laboratories, Freemont CA). Sections were blocked sequentially with avidin, biotin (Biogenex Laboratories, Freemont CA) and FC receptor blocker (Innovex Biosciences, Richmond CA) for 20 min at RT. Primary antibody to PD-L1 was applied at 5 μg/ml overnight at 4 °C. Multilink secondary antibody (Biogenex Laboratories, Freemont CA) was applied for 30 min at RT, followed by Streptavidin-labeled peroxidase (Biogenex laboratories, Fremont CA) for 30 min. The immunostaining was developed with Turbo DAB chromogen (Innovex Biosciences, Richmond CA) for 2 min or until signal appeared.

### Culture and activation of γδ T cells

Preparation and testing expanded/activated γδ T cells from healthy volunteers was performed using a modified version of a previously described method^13^. Up to 50 mL of peripheral apheresis products was obtained from healthy volunteers following informed consent and purchased from Hemacare (Van Nuys, CA). Mononuclear cells (MNC) were isolated using Ficoll and resuspended at a concentration of 1 × 10^6^ cells/mL in a 50/50 mixture of RPMI-1640 (Life Technologies; Carlsbad, CA) and Clicks EHAA (Irvine Scientific; Santa Ana, CA) media supplemented with 15% pooled human AB serum, 5 mM Zoledronic Acid (Zoledronate; Novartis; Basel, Switzerland), and 50U/mL human rIL-12 (Miltenyi Biotech; Auburn, CA). Cultures were transduced with a P140k-MGMT expressing lentivector (Miltenyi Lentigen, Gaithersburg, MD) at a multiplicity of infection (MOI) of 10 at days 5 and 6 ± 1 and maintained for 14 ± 2 days with the addition of fresh complete media as needed to maintain a total cell concentration of 1 × 10^6^/mL. Vector copy number (VCN) was determined by QRT-PCR.

### Flow cytometry

Immunophenotyping of cultures was performed on a FACS Canto II flow cytometer or LSR Fortessa SE flow cytometer (BD Biosciences; San Jose, CA) using BD Lymphocyte Immunophenotyping System and Beckman-Coulter (Miami, FL) Duraclone tubes for TCR analysis and determination of effector/memory status. List mode data were analyzed using DiVa and Cell Quest Pro software (BD Biosciences; San Jose, CA). Characterization and enumeration of lymphocytes pre- and post- expansion culture and the final infused cell product was performed using single-platform flow cytometry via surface labeling using directly conjugated monoclonal antibodies (mAbs) against CD45 (2D1), CD3 (SK7), CD4 (SK3), CD8 (SK1), CD16/56 (B73.1/NCAM 16.2), CD19 (SJ25C1), CD27 (M-T271), CD45RA (L48), TCR-γδ (11F2), Vδ1 (TS 8.2), and Vδ2 (B6). Expression of known stress-associated molecules was measured in glioma xenolines by flow cytometry after staining with antibodies that bind cell stress-induced markers (NKG2D ligands).

### Cytotoxicity assays

Cytotoxicity of expanded γδ T cells to xenolines was determined in vitro by flow cytometry using a commercial kit (Immunochemistry Technologies; Bloomington, MN) incorporating dye that labels target cells (CSFE) and a dye that labels dead cells (7-AAD). The standardized K562 erythroleukemia cell line was included in the assay as positive cytotoxicity control. Briefly, target cells were labeled with CSFE according to manufacturer’s protocol. Expanded/activated γδ T cells were then added to the tubes at ratios of 0:1 (Background), 5:1, 10:1, 20:1 effectors/targets, incubated for four hours at 37 °C and 5% CO2, washed once and resuspended in 1 ml HBSS. 7-AAD solution (20ul) was added prior to acquisition on the flow cytometer.

Percent specific release was calculated as follows:

$$\% {\text{ Specific }}\;{\text{Release}} = \frac{{{\text{fluorescence}} _{{({\text{experiment}})}} - {\text{ fluorescence }}_{{({\text{spontaneous }}\;{\text{release}})}} }}{{{\text{fluorescence}} _{{({\text{maximum}})}} - {\text{ fluorescence}} _{{({\text{spontaneous}}\;{\text{ release}})}} }} \times 100$$

## Supplementary Information

Supplementary Information 1.

Supplementary Information 2.
