# Supplementary material for: A combined treatment regimen of MGMT-modified γδ T cells and temozolomide chemotherapy is effective against primary high grade gliomas
Source: Sci Rep. 2021 Oct 26;11:21133. doi: 10.1038/s41598-021-00536-8 (PMC8548550; doi:10.1038/s41598-021-00536-8)
Supplement: Supplementary file 1 — Supplementary Information 1. [file 41598_2021_536_MOESM1_ESM.docx]

**Legends for Supplemental Figures**

Figure S1: (a) Stress-associated NKG2DL expression on JX12T and JX22T glioma xenolines. PMT voltage is not adjusted for autofluorescence seen on both xenolines. Black = isotype control; blue = xenolines + growth media, red = xenolines + growth media containing 400μM TMZ. Flow cytometry was performed 4h following initiation of culture. Note expression of MIC-A, MIC-B, ULBP-1 and ULBP-4 on JX12T and shows slight upregulation of ULBP-1 and ULBP-4 following TMZ exposure. JX22T expresses MIC-A, MIC-B, ULBP-1 and ULBP-4 , showing TMZ-induced upregulation of MIC-A and ULBP-4. (b) ULBP-4 expression on JX59T xenograft following 7 days of growth in an untreated mouse and (c) four hours following intraperitoneal injection of 60mg/kg TMZ. Note lighter increased intensity of ULBP-4 expression on the tumor following exposure to TMZ.
