# Supplementary figures and images for: A combined treatment regimen of MGMT-modified γδ T cells and temozolomide chemotherapy is effective against primary high grade gliomas
Source: Sci Rep. 2021 Oct 26;11:21133. doi: 10.1038/s41598-021-00536-8 (PMC8548550; doi:10.1038/s41598-021-00536-8)

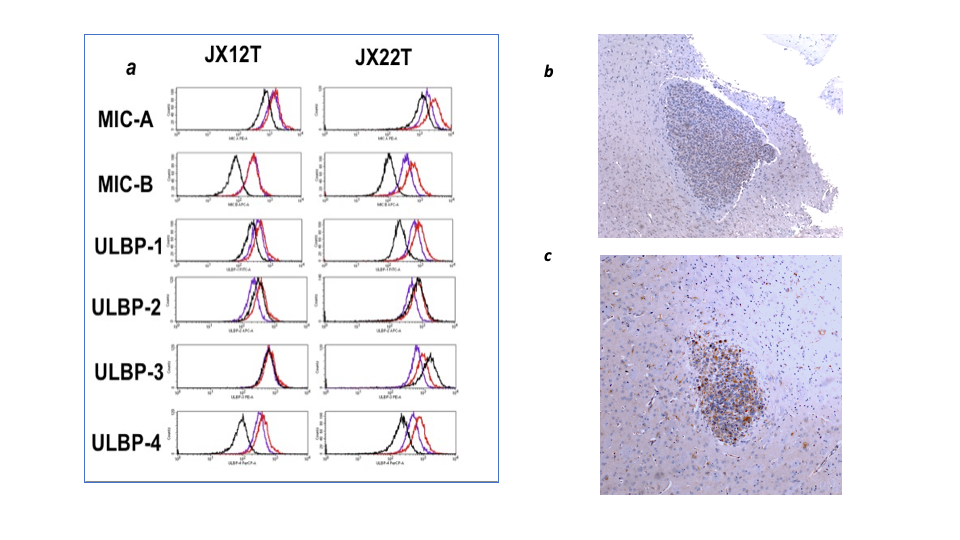

Supplement: Supplementary file 2 — Supplementary Information 2. [file 41598_2021_536_MOESM2_ESM.tiff]
